# Effect of water management on microbial diversity and composition in an Italian rice field system

**DOI:** 10.1093/femsec/fiac018

**Published:** 2022-02-16

**Authors:** Eric R Hester, Annika Vaksmaa, Giampiero Valè, Stefano Monaco, Mike S M Jetten, Claudia Lüke

**Affiliations:** Department of Microbiology, IWWR, Radboud University Nijmegen, Nijmegen, 6525 AJ, The Netherlands; Laboratory of Microbiology, Wageningen University and Research, Wageningen, 6700 EH, The Netherlands; Department of Microbiology, IWWR, Radboud University Nijmegen, Nijmegen, 6525 AJ, The Netherlands; Department of Marine Microbiology and Biogeochemistry, NIOZ Royal Institute for Sea Research, Utrecht University, 1790 AB Den Burg, Texel, The Netherlands; CREA - Council for Agricultural Research and Economics, Research Centre for Cereal and Industrial Crops, 13100, Vercelli, Italy; DiSIT-Dipartimento di Scienze e Innovazione Tecnologica, Università del Piemonte Orientale, Piazza San Eusebio 5, I-13100 Vercelli, Italy; CREA - Council for Agricultural Research and Economics, Research Centre for Cereal and Industrial Crops, 13100, Vercelli, Italy; CREA - Council for Agricultural Research and Economics, Research Centre for Engineering and Agro-food processing, Strada delle Cacce 73, 10135 Torino, TO, Italy; Department of Microbiology, IWWR, Radboud University Nijmegen, Nijmegen, 6525 AJ, The Netherlands; Soehngen Institute of Anaerobic Microbiology, Nijmegen, The Netherlands; Department of Microbiology, IWWR, Radboud University Nijmegen, Nijmegen, 6525 AJ, The Netherlands

**Keywords:** archaea, bacteria, bulk soil, differential abundance, diversity, methane, paddy, roots

## Abstract

Traditional rice cultivation consumes up to 2500 L of water per kg yield and new strategies such as the ‘Alternate Wetting and Drying’ (AWD) might be promising water-saving alternatives. However, they might have large impacts on the soil microbiology. In this study, we compared the bacterial and archaeal communities in experimental field plots, cultivated under continuously flooding (CF) and AWD management, by high-throughput sequencing of the 16S rRNA gene. We analysed alpha and beta diversity in bulk soil and on plant roots, in plots cultivated with two different rice cultivars. The strongest difference was found between soil and root communities. Beside others, the anaerobic methanotroph *Methanoperedens* was abundant in soil, however, we detected a considerable number of ANME-2a-2b on plant roots. Furthermore, root communities were significantly affected by the water management: Differential abundance analysis revealed the enrichment of aerobic and potentially plant-growth-promoting bacteria under AWD treatment, such as *Sphingomonadaceae* and *Rhizobiaceae* (both *Alphaproteobacteria*), and *Bacteroidetes* families. Microorganisms with an overall anaerobic lifestyle, such as various *Delta*- and *Epsilonproteobacteria*, and *Firmicutes* were depleted. Our study indicates that the bulk soil communities seem overall well adapted and more resistant to changes in the water treatment, whereas the root microbiota seems more vulnerable.

## Teaser

This study evaluates the effect of water-saving rice cultivation on the paddy field microbiota, showing that root-associated microorganisms seem more vulnerable to the different water management than the bulk soil microbiota.

## Introduction

Rice (*Oryza sativa* L.) is an important crop and staple food for more than half of the world's population. It is cultivated on over 160 million hectares of land world-wide and production needs to further increase to meet the demands of the growing human population (Van Nguyen and Ferrero 2006). Irrigated rice, in which fields are permanently covered with water by artificial irrigation, is the most productive agricultural practice to date and accounts for approximately 75% of all rice yields (Rao et al. 2017). With that, rice agriculture forms the largest water consumer in the agricultural sector (Thakur et al. 2014). Ongoing climate change and associated drought and heat events are leading to reduced availability of freshwater and thus, efficient and sustainable water management strategies are urgently needed. In addition to water consumption, flooded rice fields significantly contribute to the emission of the greenhouse gas methane, accounting for approximately 8% to the total global anthropogenic emission (Saunois et al. 2020). An adaption of rice cultivation strategies towards aerobic rice cultivation or intermittent flooding might not only save water, but also reduce methane emissions to the atmosphere. However, cultivation of rice under aerobic conditions faces new challenges and is often associated with reduced yields and problems in pest control (Kreye et al. 2009, Price et al. 2013, Volante et al. 2017). One of the more recent and promising approaches towards a more sustainable rice agriculture is a water management referred to as ‘Alternate Wetting and Drying’ (AWD) (Price et al. 2013). It combines the beneficial aspects of aerobic and anaerobic cultivation and implies an initial flooding of the fields, followed by a period in which fields are allowed to dry to a defined water level, and are then re-flooded again. Studies have shown that this technique is efficient in water reduction and can result in even higher yields and nutritional status than conventional flooded cultivation (Yang et al. 2009, Carrijo et al. 2017). It can also reduce methane emissions to the atmosphere by 35%–90% (Chu et al. 2015, Yang et al. 2017, Chidthaisong et al. 2018, Linquist et al. 2018, Setyanto et al. 2018, Tran et al. 2018). However, more research is needed to demonstrate whether this strategy is generally applicable, and if it provides a viable alternative in all geographical regions. Studies evaluating AWD managements in European rice agriculture for instance are still limited to date (Oliver et al. 2019, Monaco et al. 2021).

To assess the impacts of alternative water management practice on climate and on crop growth yields, it is crucial to understand the underlying microbiology in these systems. Microorganisms play a key role in rice field ecology as they are responsible for cycling and availability of nutrients, as well as the emission of greenhouse gases such as methane, CO_2_, and nitrous oxide (N_2_O). Furthermore, some beneficial microorganisms can interact with the rice plant in a growth promoting manner (Berg et al. 2014). Under conventional, permanently flooded cultivation, the biogeochemistry of rice fields is largely controlled by the restricted availability of oxygen (Frenzel and Conrad 2003). After flooding, the vast amount of the bulk soil rapidly becomes anoxic. The complex microbial communities thriving in these soils then widely consists of (facultatively) anaerobic microorganisms that use alternative electron acceptors such as nitrate, ferric iron and sulfate. Furthermore, fermenting microorganisms that are disproportionating organic substrates, as well as syntrophic microorganisms that produce H_2_, are playing key roles (Frenzel and Conrad 2003 and references therein). Methane is formed as an end product of the anaerobic degradation of organic matter by methanogenic archaea, after the alternative electron acceptors are depleted. Nevertheless, aerobic niches also persist in flooded soils, specifically in the top layer of the surface soil and in the rhizosphere (Frenzel et al. 1992, Revsbech et al. 1999). Here, redox-cycling takes place and electron acceptors are regenerated (e.g. Begg et al. 1994, Wind and Conrad 1995). Part of the methane is also oxidized by aerobic or anaerobic microorganisms before reaching the atmosphere (e.g. Wassmann and Aulakh 2000, Fan et al. 2021). Despite their central role in paddy field ecology, there is a lack of studies holistically investigating the effect of alternative water managements on bacterial and archaeal communities in paddy fields. Here, we performed high-throughput amplicon sequencing of the 16S rRNA gene and analysed the diversity of Bacteria and Archaea under conventional flooding (CF) and AWD water management in an Italian rice field ecosystem. In a complementary part of this study, the general plant performance was evaluated demonstrating that the AWD treatment has substantial water saving potential and only very limited effects on crop status and final productivity (Monaco et al. 2021).

As the flooding of rice fields largely controls the oxygen availability and biogeochemistry within the ecosystem, we hypothesized that an alternate water management will effect the composition of microorganisms in the fields. To address this question, we investigated the overall alpha and beta-diversity in the two main field compartments (bulk soil versus rice roots). To further test if microorganisms are differently effected in fields planted with different rice varieties, we selected plots cultivated with the two varieties *O. sativa japonica* cultivar Centauro and *O. sativa japonica* cultivar Vialone Nano. To analyse the water management effect on individual microbial groups in more detail, we performed differential abundance analysis.

## Materials and methods

### Experimental design and sampling

The field site was located at the CREA—Research Centre for Cereal and Industrial Crops in Vercelli, Italy (45°19'21.96'' N, 8°22'24.07'' E). More detailed information on soil characteristics and climate is described in Oliver et al. (2019) and Monaco et al. (2021). The experiment was set up in 2015 and consisted of four replicated field blocks, each block contained one site under continuously flooded water management (CF) and one site under AWD management (Fig. S1). Each treatment was further divided into individual plots (1.6×5m) planted with 12 different European rice cultivars in randomised order. The plots were fertilized pre-sowing in April with commercial dry manure (260 kg ha-1, total N content: 12.5%). Top dress fertilization was added in June (300 kg ha-1). The AWD water management was applied in the vegetative growth cycle of the plants (tillering, stem elongation). After dry seeding in May, all plots were covered with water to 5 cm above soil surface in June, when rice was at three leaves phase. The plots under AWD treatment were then allowed to naturally dry out while the plots under CF treatment were kept continuously flooded. Whenever the soil matric water potential reached –30 kPa (at 25 cm depth) in the AWD treatment, the plots were re-flooded and allowed to dry out afterwards again. See Oliver et al. (2019) and Monaco et al. (2021) for a more detailed description of agricultural practice and water management. In this study, sampling of soil and plant roots took place begin August 2015, during initiation of the panicle formation in the rice plants (R0 stage of the rice growth stages; Counce et al. 2000) and before all plots were continuously flooded for plant flowering. The two rice cultivars with the most different growth phenotypes were chosen: *O. sativa japonica* cultivar Vialone Nano and *O. sativa japonica* cultivar Centauro. They belong to two different mereological classes, round (Centauro) and medium (Vialone Nano), and are substantially different based on a phylogenic analysis using 9.996 random Single Nucleotide Polymorphism (SNP) markers (Volante et al. 2017). From all plots planted with these two cultivars (4 under AWD and four under CF; Fig. S1), soil samples of the top 20 cm were taken with a soil corer (three samples per plot at random places in-between the plants) and rice plants were carefully harvested without destroying the roots (three randomly selected plants per plot). Roots were gently washed in a bucket with clean tap water to remove the excess soil, cut with scissors and packed into sterile 50 ml falcon tubes. Our sampling strategy does not discriminate between microorganisms attached to the root surface (rhizoplane) and the microorganisms in internal tissues of the roots (endosphere). All samples were stored at 4°C immediately after collection and during transportation to the laboratory, where they were frozen at –20 °C until DNA extraction and further processing.

### DNA extraction and amplicon sequencing

DNA extraction from 0.25 to 0.75 g soil or roots samples was performed in duplicate with the PowerSoil DNA isolation Kit (Mo bio Laboratories Inc., Carlsbad, USA) according to the manufacturer's protocol. Roots were ground in liquid nitrogen with mortar and pestle prior to extraction. DNA quantity was assessed by UV-VIS spectroscopy (Nanodrop, ND-1000, Isogen Life Science, the Netherlands). DNA from the extracted samples was pooled in equimolar amounts. DNA was sequenced targeting the v3-v4 region of the 16S rRNA gene at the Macrogen Europe B.V. Bacteria and Archaea were targeted using the primer pairs 341F-785R and 349F-786R, respectively (Takai and Horikoshi 2000, Klindworth et al. 2013).

### Data analysis

High-throughput sequencing of the bacterial and archaeal 16S rRNA genes revealed a total of 8554672 and 7185692 unpaired reads, respectively. 16S rRNA gene reads were paired using pandaseq with default settings (Masella et al. 2012). Quality control was performed using prinseq (Schmieder and Edwards 2011). Paired reads were filtered for a mean quality (Q > 25) and size (> 250 bp). Pairing and QC resulted in 3493820 archaeal and 3377035 bacterial reads. Quality controlled reads were then used in the NINJA-OPS v1.3 pipeline (Al-Ghalith et al. 2016) with default settings and the SILVA database version 123 (Quast *et al*. 2012; Yilmaz e*t al*. 2014) as reference for taxonomic assignment (97% identity). The biom formatted OTU (Operational Taxonomic Units) table was converted into a legacy format (McDonald et al. 2012) and all downstream analysis was performed on this OTU table in R version 4.0.3 (R Core Team 2020). Alpha- and beta-diversity analysis was carried out using the ‘pyloseq’ package version 1.34.0 and the ‘vegan’ package version 2.5–6 implemented in R (McMurdie and Holmes 2013, Oksanen et al. 2019). Graphs were created using the ‘ggplot2’ package version 3.3.2 (Wickham 2016). First, data was filtered based on taxonomy and OTUs assigned to Archaea/Eukaryota were removed from the bacterial dataset and Bacteria/Eukaryota were removed from the archaeal dataset. In the bacterial dataset, an additional OTU not assigned to any phylum, and OTUs assigned to Chloroplast and Mitochrondria were removed. Alpha diversity indices were calculated using the ‘estimate_richness’ function in ‘phyloseq’ and the Wilcoxon rank-sum test (‘wilcox.test’ function) was performed to test for significant differences between alpha diversity measures of different groups (*P* <0.05). Prior NMDS analysis, data was further filtered based on prevalence and all OTUs occurring in less than 2% of all samples were removed. Abundance values were transformed to median sampling depth. NMDS was performed using the ‘metaMDS’ function with Bray–Curtis distance and 1000 maximum random starts to find a stable solution. Analysis of similarities (‘anosim’ function) was used to test for significant differences between groups.

Differential abundance analysis was performed using the DESeq2 package version 1.30.0 (Love et al. 2014). Analysis was done on taxonomy filtered, raw read counts and for the archaeal dataset, samples with less than 1500 reads were further removed prior to analysis. Root and Soil samples were treated separately. The phyloseq data was converted to the relevant DESeqDataSet object using the ‘phyloseq_to_deseq2’ function. Differential abundance analysis was then performed using the ‘DESeq’ and ‘results’ function and the significant cut-off in optimizing the independent filtering (alpha) was set to 0.01. Graphs were created using the ‘ggplot2’ package version 3.3.2 (Wickham 2016).

### Data availability

Raw sequences were submitted to the NCBI Sequence Read Archive (SRA) and are available under the BioProject ID PRJNA777437.

## Results

### Microbial communities in the rice field ecosystem: alpha and beta diversity

High-throughput sequencing of the bacterial and archaeal 16S rRNA genes revealed, after taxonomic filtering, a total of 932612 archaeal and 1603281 bacterial reads, resulting in a total of 22544 bacterial and 719 archaeal OTUs.

**Figure 1.**
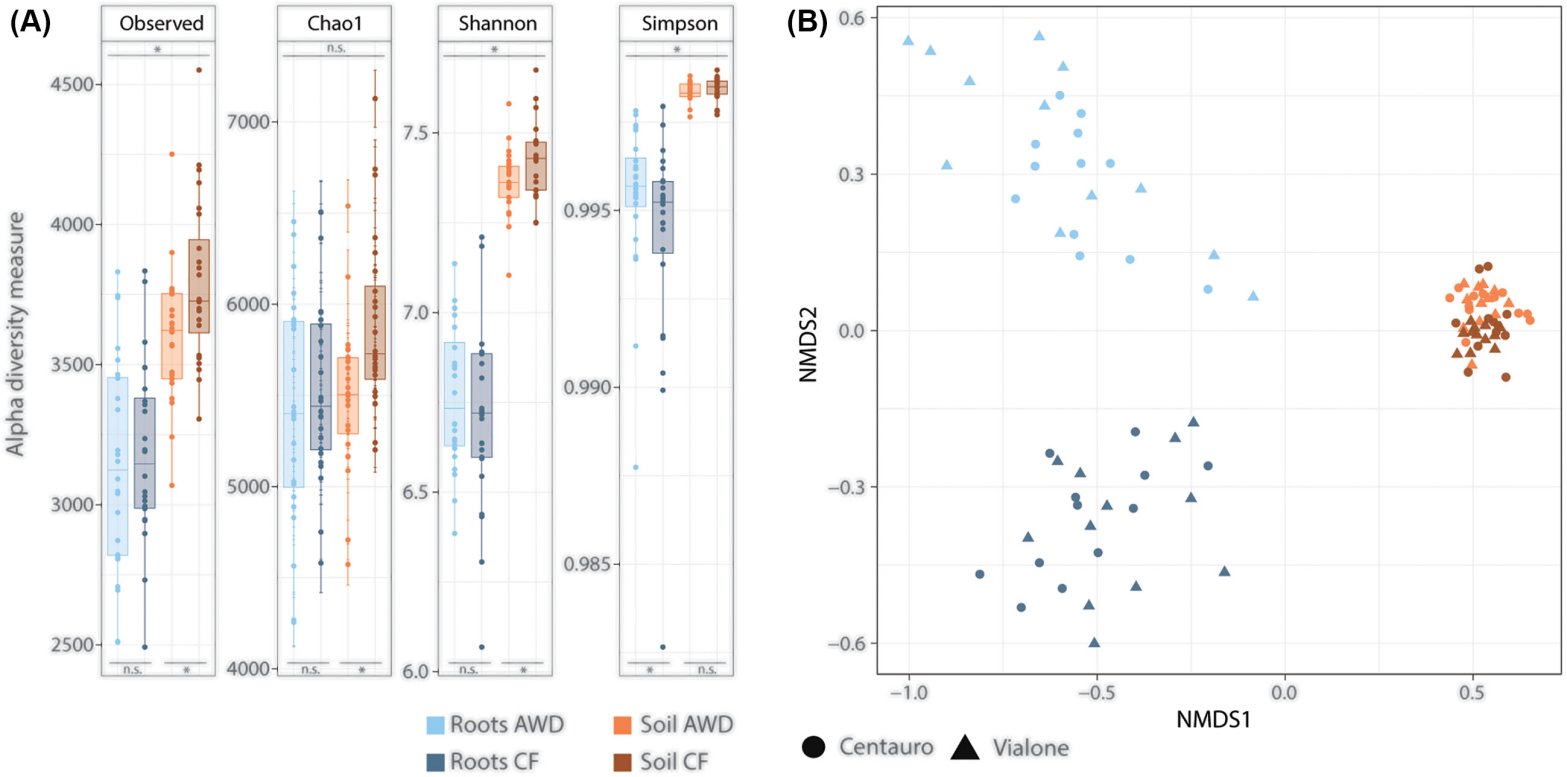
Bacterial diversity in paddy soil samples and on roots of rice plants under different water management (CF = Continuously Flooded, AWD = Alternate Wetting and Drying). **(A)** Alpha diversity measured by different diversity indices: observed species richness (Observed), estimated species richness (Chao1), Shannon index (Shannon), and Simpson index (Simpson). The asterisk indicates significant differences between groups according to Wilcoxon rank-sum test (*P* <0.05, n.s. = not significant). Top: test between root and soil, Bottom: test between water management. **(B)** Beta diversity analysed by non-metric multidimensional scaling (NMDS). In addition to the sampled compartment (roots or soil) and water management (CF and AWD), the rice variety planted on individual plots is shown (Vialone Nano and Centauro). (NMDS stress = 0.0699).

**Figure 2.**
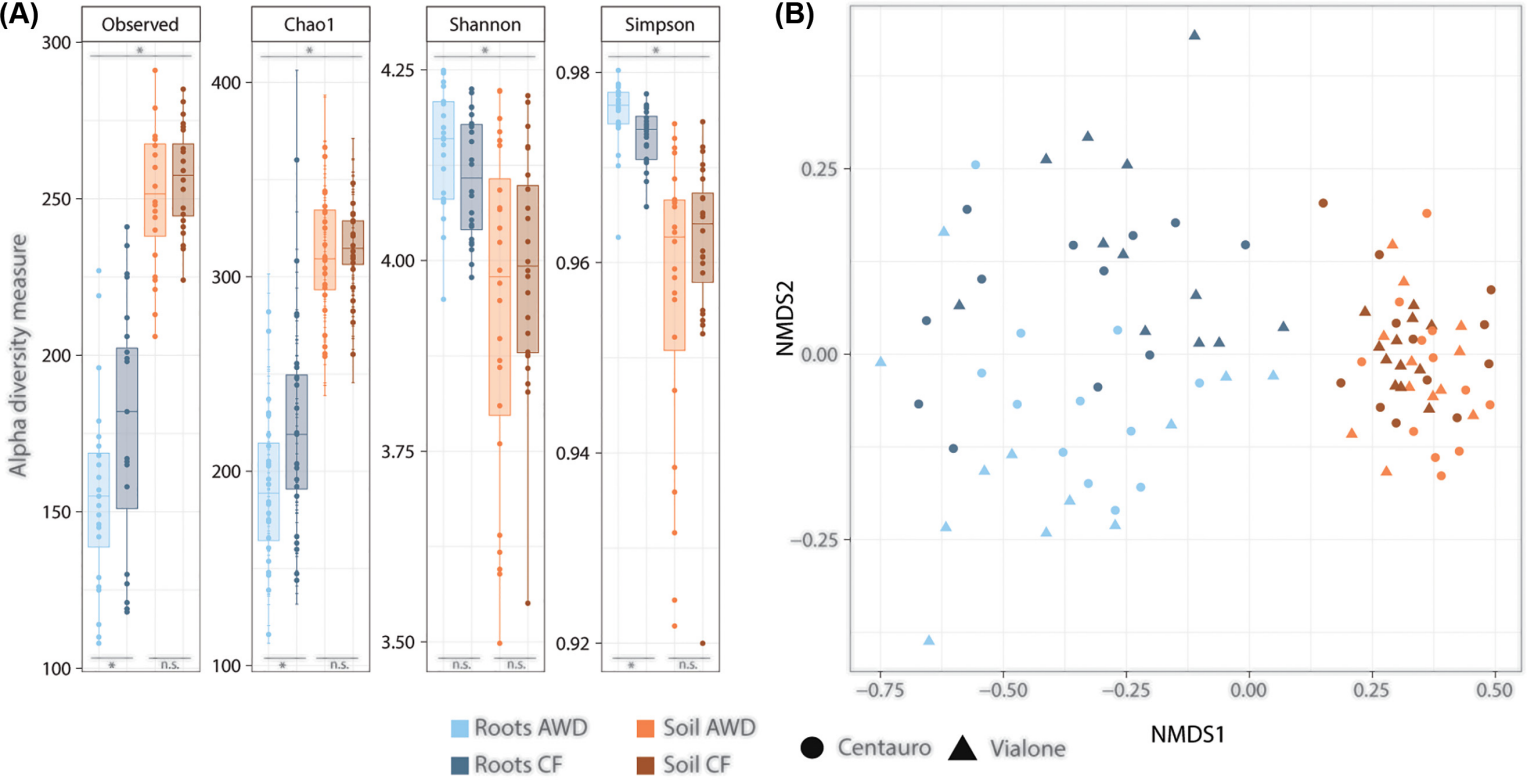
Archaeal diversity in paddy soil samples and on roots of rice plants under different water management (CF = Continuously Flooded, AWD = Alternate Wetting and Drying). **(A)** Alpha diversity measured by different diversity indices: observed species richness (Observed), estimated species richness (Chao1), Shannon index (Shannon), and Simpson index (Simpson). The asterisk indicates significant differences between groups according to Wilcoxon rank-sum test (*P* <0.05). Top: test between root and soil, Bottom: test between water management (no sig. = no significance). **(B)** Beta diversity analysed by non-metric multidimensional scaling (NMDS). In addition to the sampled compartment (roots or soil) and water management (CF and AWD), the rice variety planted on individual plots is shown (Vialone Nano and Centauro). (NMDS stress = 0.1).

The observed number of OTUs (species richness) in the individual samples was significant higher in soil than in root samples (Fig. 1A, Fig. 2A). This was the case for bacteria as well as archaea (Wilcoxon rank sum test; bacterial OTUs: W = 290.5, *P* <0.05; archaeal OTUs: W = 41, *P* <0.05). Nevertheless, the Chao1 index only estimated significantly higher OTU richness in soil samples for archaea (W = 87, *P* <0.05). Shannon and Simpson indices also revealed a significantly higher bacterial diversity in soil samples (Shannon: W = 3, *P* <0.05; Simpson: W = 10, *P* <0.05), whereas the archaeal diversity was significantly higher on plant roots (Shannon: W = 1784, *P* <0.05; Simpson: W = 2171, *P* <0.05). Species diversity as measured by the Shannon and Simpson indices can be divided into two components: species richness and evenness. Archaeal richness (Observed number of OTUs and Chao1 index; see above) was higher in soil than in root samples. However, Shannon and Simpson diversity was higher in root samples. This indicates a lower richness, but a more even distribution of species abundance (higher evenness) for the archaea on plant roots compared to the soil.

To test whether the water management had a significant influence on the microbial diversity, root and soil samples were analysed separately. No significant difference was found for the bacterial diversity in the root samples, but the soil samples showed a higher richness and diversity in soils under continuous flooding (CF) compared to AWD (Fig. 1A) (Observed richness: W = 176, *P* <0.05; Chao1: W = 156, *P* <0.05; Shannon: W = 185, *P* <0.05; Simpson: not significant, p>0.05). For the archaea, an effect of water management could be detected in the roots samples. Here, higher archaeal richness was again seen under CF treatment (Fig. 2A) (Observed: W = 180.5, *P* <0.05; Chao1: W = 189, *P* <0.05). Nevertheless, Shannon and Simpson indices showed again the opposite trend as the richness indices, revealing a higher diversity under AWD treatment (Shannon: not significant, p>0.05, Simpson: W = 441, *P* <0.05). Thus, whereas the overall archaeal richness was higher under CF treatment, species abundances seemed to be more evenly distributed under AWD management. The soil samples showed no significant effect of water management on the archaeal diversity (Fig. 2A). Furthemore, no significant differences in microbial diversity could be observed between plots planted with the two rice cultivars (Fig. S2).

To further compare the overall microbial composition in the field plots, non-metric multidimentional Scaling (NMDS) analysis was performed. The NMDS analysis revealed a clear separation of root and soil samples along the first axis, based on both, bacterial and archaeal OTU composition (Figs 1B and 2B). The separation was more pronounced for the bacteria. Analysis of similarities (ANOSIM) supported a significant clustering of root and soil samples for both data sets, respectively (Bacteria: R = 0.994, *P* = 0.001; Archaea: R = 0.897, *P* = 0.001). A further separation of root samples according to water management could be observed along the second NMDS axis. This effect was more distinct in the analysis of bacteria (Fig. 1B) (ANOSIM R = 0.846, *P* = 0.001). In fact, a grouping of the soil samples according to water management was not supported by ANOSIM (R = 0.121, *P* = 0.002). Also for the archaeal dataset, a weak gradient separation of root samples according to water management could be observed in NMDS analysis (Fig. 2B), however, a strong distinct grouping was not supported by ANOSIM (R = 0.267, *P* = 0.001).

The clear separation of root and soil samples, revealed by NMDS, was also reflected by different community compositions at the phylum level (Figs S3 and S4). Root samples were characterized by a strong dominance of *Proteobacteria*, contributing approximately half to the overall bacterial composition. Furthermore, *Bacteroidetes* (ca. 15%), *Actinobacteria* (ca. 10%) and *Firmicutes* (ca. 6%) formed highly abundant phyla. Soil samples were also dominated by *Proteobacteria*, however, to a less amount (ca. 29%). In contrast to roots, the soil contained a high relative abundance of *Acidobacteria* (ca. 21%). In addition, *Bacteroidetes* (ca. 7%), *Chloroflexi* (ca. 7%), *Planctomycetes* (ca. 6%), *Verrucomicrobia* (ca. 6%) and *Nitrospirae* (ca. 6%) were abundantly present. The archaeal communities were dominated by three main phyla: *Euryarchaeota*, the *Miscellaneous Crenarchaeotic Group* (*Bathyarchaeota*) and *Thaumarchaota*. Whereas root samples were highly dominated by *Euryarchaeota* (ca. 62%), soil samples contained a more even distribution of all three phyla (Fig. S4). Within *Euryarchaeota*, the methanogenic families *Methanocellaceae*, *Methanobacteriaceae*, *Methanosaetaceae* and *Methanosarcinaceae* formed the largest families on rice roots (together ca. 48% of the total archaea). Anaerobic methanotrophs of the *GOM Arc I* group (including *Methanoperedens*) and *ANME-2a-2b* were also present, but in less abundance (ca. 7% of the total archaea). In contrast, *Methanoperedens*-like methanotrophs dominated the *Euryarchaeota* in soil samples (ca. 29% of all *Euryarchaeota*, ca. 10% of all archaea), followed by methanogens of the families *Methanosaetaceae*, *Methanocellaceae, Methanosarcinaceae*, and *Methanobacteriaceae*. Interestingly, the *ANME-2a-2b* group was nearly absent in the soil. Abundant thaumarchaeotic classes in all samples were the *Soil Crenarchaeotic Group* (*SCG*), the *South African Gold Mine Gp1* (*SAGMCG-1*) and the *Marine Group I*. Root samples were dominated by the *SCG* and soil samples by the *SAGMCG-1*. *SCG* and *SAGMCG-1* comprise, besides others, well characterized ammonia oxidizing archaea of the genera *Nitrososphaera* and *Nitrosotalea*, respectively. The *Marine Group I* includes the ammonia oxidizers *Nitrosopumilus*, *Nitrosoarchaeum* and *Nitrosopelagicus*.

### Effect of water management: differential abundance analysis

**Figure 3.**
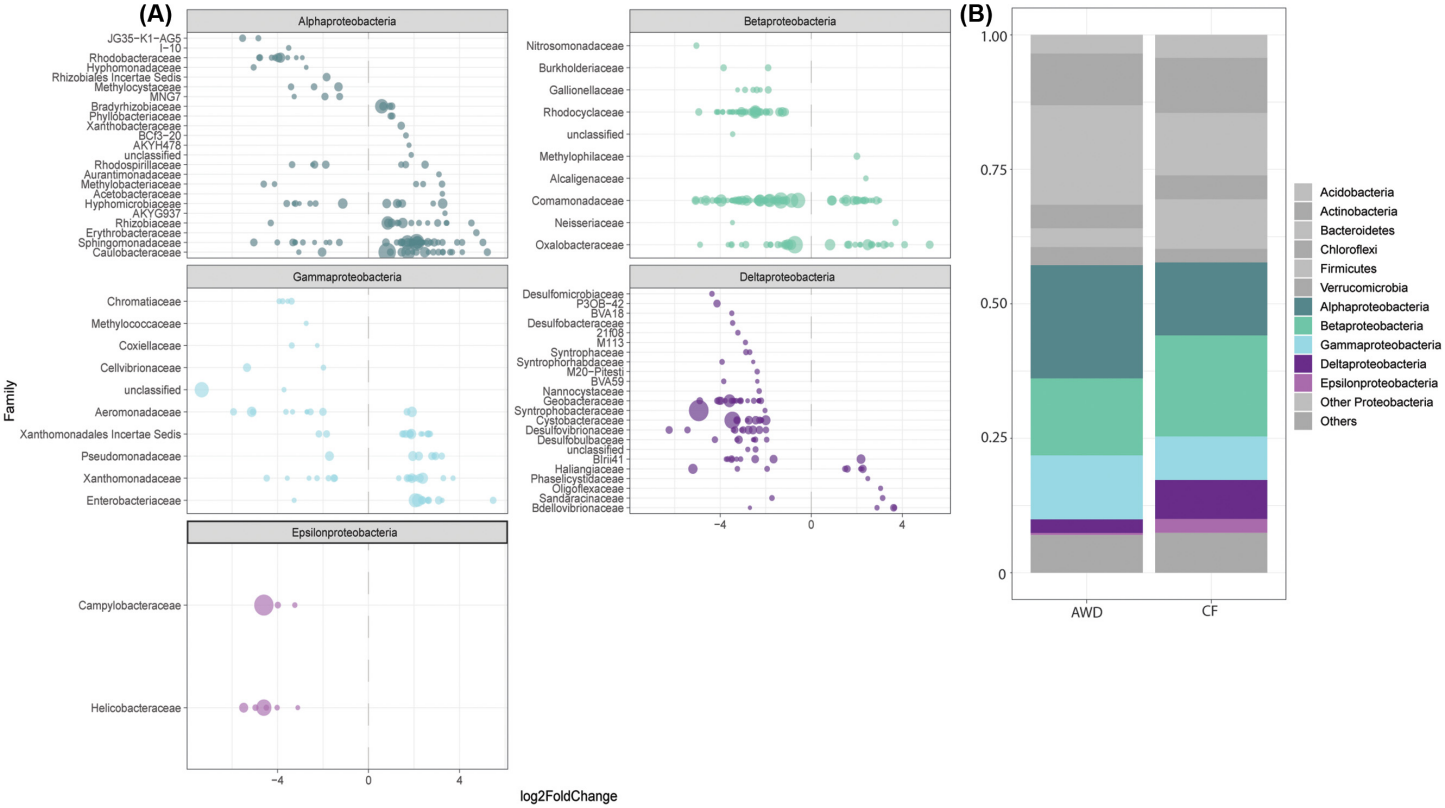
Effect of water management on Proteobacteria in roots samples. **(A)** Log_2_-fold changes of differentially abundant OTUs in AWD (Alternate Wetting and Drying) versus CF (Continuously Flooded) treatment (adjusted *P*-value cutoff = 0.01). CF treatment was defined as control. OTUs were grouped on the family level. Circle sizes are correlated to the baseMean (mean of normalized counts for all samples) of the respective OTU. **(B)** Mean relative abundances of total OTUs in root samples (n = 17722) under AWD and CF treatment.

To get more insights into the effect of water management on the microbial composition, we performed differential abundance analysis of OTUs in samples under AWD versus CF treatment (defined as control). Prior analysis, we separated the data according to root and soil samples. Analysis of bacterial OTUs in root samples revealed 1075 OTUs (out of in total 17722 OTUs) that were significantly changed in abundance, of which 405 OTUs were more abundant and 670 OTUs less abundant under AWD treatment (adjusted *P*-value cutoff = 0.01). Most of these OTUs (n = 478) were classified as *Proteobacteria* (Fig. 3A). As described above (see Fig. S3), *Proteobacteria* also formed the largest bacterial phylum in root samples. Comparing the mean relative abundance of *Proteobacteria* under the different water management revealed no changes at the phylum level, however changes could be observed at the class level (Fig. 3B). Whereas the overall relative abundance of *Alpha-* and *Gammaproteobacteria* increased under AWD treatment, *Beta-*, *Delta-* and *Epsilonproteobacteria* decreased. Differential abundance analysis at the OTU level revealed a more complex pattern (Fig. 3A): the majority of significantly affected alpha- and gammaproteobacterial taxa were indeed more abundant, nevertheless, several OTUs were also less abundant. Ambiguous patterns could be even observed for taxa of the same family. However, OTUs with a high overall abundance across samples (represented by larger circle sizes in Fig. 3A), such as taxa of the alphaproteobacterial families *Bradyrhizobiaceae*, *Rhizobiaceae*, *Sphingomonadaceae*, and *Caulobacteraceae* were significantly increased in AWD treatment. Within the *Gammaproteobacteria*, *Enterobacteriaceae* and *Xanthomonadaceae* belonged to the dominant families affected by water management, also with higher abundance under AWD treatment (Fig. 3A). Dominant betaproteobacterial taxa, such as the *Rhodocyclaceae* and *Comamonadaceae*, were mostly less abundant in AWD treatment. Nevertheless, ambiguous patterns could be observed for taxa within the *Oxalobactericeae* and also the *Comamonadaceae*. For the *Delta-* and *Epsilonproteobacteria*, the vast majority or even all taxa were less abundant under AWD treatment (Fig. 3A).

**Figure 4.**
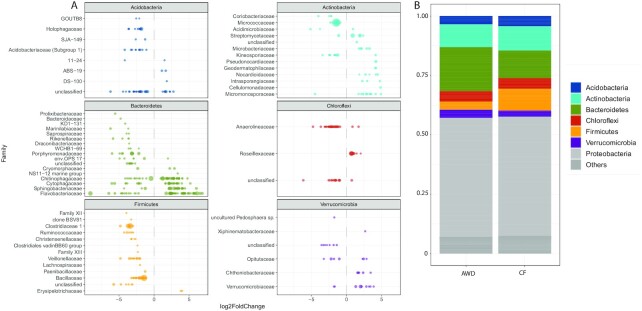
Effect of water management on dominant bacterial phyla in roots samples. Less dominant phyla are shown in Figure S5. **A**. Log_2_-fold changes of differentially abundant OTUs in AWD (Alternate Wetting and Drying) versus CF (Continuously Flooded) treatment (adjusted p-value cutoff = 0.01). CF treatment was defined as control. OTUs were grouped on the family level. Circle sizes are correlated to the baseMean (mean of normalized counts for all samples) of the respective OTU. **B**. Mean relative abundances of total OTUs in root samples (n = 17722) under AWD and CF treatment.

Figure 4 shows the effect of the water management on the remaining bacterial phyla in root samples. OTUs of the rare phyla in root samples are summarized in Fig. S5. The clearest distribution could be observed within the *Firmicutes* (Fig. 4A and Fig. S7). Here, nearly all taxa showed significantly less abundance under AWD treatment. This is also reflected by the strong decrease of the total *Firmicutes* in mean relative abundance (Fig. 4B). In contrast, the *Bacteroidetes* strongly increased in relative abundance. At the OTU level, however, this distribution is more ambiguous. Of the significantly affected *Bacteroidetes*, 104 taxa were more abundant and 76 taxa less abundant in AWD treatment. Within the *Chloroflexi*, a differentiation between families could be seen: OTUs of the *Anaerolineaceae* increased and OTUs of the *Roseiflexaceae* decreased under AWD treatment.

**Figure 5.**
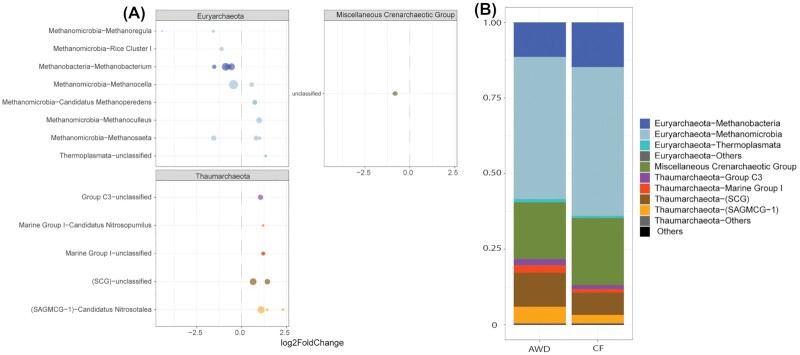
Effect of water management on Archaea in roots samples. **A**. Log_2_-fold changes of differentially abundant OTUs in AWD (Alternate Wetting and Drying) versus CF (Continuously Flooded) treatment (adjusted *P*-value cutoff = 0.01). CF treatment was defined as control. OTUs were grouped on the family level. Circle sizes are correlated to the baseMean (mean of normalized counts for all samples) of the respective OTU. **B**. Mean relative abundances of total OTUs (n = 538) under AWD and CF treatment.

Differential abundance analysis of archaeal OTUs within root samples revealed 28 taxa (out of in total 538 OTUs) that significantly changed in abundance (adjusted p-value cutoff = 0.01). Of these OTUs, 16 taxa increased and 12 taxa decreased in field plots under AWD treatment (Fig. 5). Whereas the OTUs of the *Miscellaneous Crenarchaeotic Group* (n = 2) decreased, all affected *Thaumarchaeota* (n = 9) showed an increase in abundance. This increase is also reflected by the increase of the overall relative abundance of *Thaumarchaeota* under AWD treatment (Fig. 5B). Within the *Euryarchaota*, 7 taxa were more abundant and 10 taxa less abundant under AWD treatment. For the soil samples, differential abundance analysis was only performed on bacterial OTUs. In fact, no significantly affected archaeal OTUs could be retrieved with the adjusted *P*-value cutoff of 0.01. This is in agreement with the NMDS analysis that also showed no separation according to water management (see above, Fig. 2B). Even for the bacterial dataset, the separation was only weak and not confirmed by ANOSIM (see above). Here, differential abundance analysis resulted in only 21 OTUs (out of totally 15781 OTUs) that were significantly affected by the water treatment (adjusted p-value cutoff = 0.01) (Fig. S6A). Accordingly, no pronounced changes could be observed between the mean relative abundance of phyla in the two treatments (Fig. S6B). Nevertheless, a weak increase of *Alphaproteobacteria* and weak decrease of *Firmicutes* and *Epsilonproteobacteria* could be seen (Fig. S6B). These trends are similar to changes observed in root samples. In fact, nearly all changes of OTUs in soil samples could be traced back in root samples, such as the increase of *Sphingomonadaceae* (*Alphaproteobacteria*), *Verrucomicrobiaceae* and *Chthoniobacteraceae* (*Verrucomicrobia*), and the decrease of *Helicobacteraceae* (*Epsilonproteobacteria*), *Rhodocyclaceae* and *Gallionellaceae* (*Betaproteobacteria*), and *Porphyromonadaceae* (*Bacteroidetes*) (Fig. S6A; Figs 3 and 4).

## Discussion

### Overall diversity in field plots

Our study revealed clear differences of the microbial composition in the bulk soil and the microbiome of the rice roots. This has been shown repeatedly (e.g. Lüke et al. 2010, Edwards et al. 2015, Breidenbach et al. 2016), and is also not surprising as plant roots alter the physico-chemical properties of their immediate surroundings. In flooded paddy fields, rice roots shape their environment in particular by the release of metabolites and oxygen into the surrounding anoxic soil (Liesack et al. 2000, Frenzel and Conrad 2003). Root-associated microbes are important for plants as they can play substantial roles in growth promotion and disease control (Lugtenberg and Kamilova 2009, Philippot et al. 2013). The rice rhizosphere was shown to enrich for a subset of microbes relative to the bulk soil, followed by an acquisition of microbial taxa on the root surface (rhizoplane) and endosphere (Edwards et al. 2015). This was also reflected by an overall decrease of microbial diversity towards the endosphere (Edwards et al. 2015). In our study, we also observed a higher OTU richness and bacterial diversity in the bulk soil compared to the rice roots, indicating the accumulation of a specialized microbial community on the roots. In particular, the increase of *Proteobacteria* and *Bacteroidetes*, and the depletion of *Acidobacteria*, *Gemmatimonadetes* and *Nitrospirae* on the roots seems to be a more general observation and was also found in other studies investigating the rice root microbiota (Edwards et al. 2015, Santos-Medellin et al. 2017, Chialva et al. 2020), and on roots of other plants species (e.g. Bulgarelli et al. 2012). Many *Proteobacteria* are fast growing organisms and might be considered as r-strategists, thriving under fluctuating, nutrient-rich conditions like those found in the rhizosphere (Philippot et al. 2013). *Proteobacteria* were also the major bacterial group incorporating photosynthetically assimilated carbon derived from rice roots (Hernandez et al. 2015) and many species are known to have plant-growth-promoting properties (Lugtenberg and Kamilova 2009, Vacheron et al. 2013). *Bacteroidetes*, in particular *Flavobacteriaceae*, are also prominent members of the plant microbiome. They are able to degrade plant-derived polysaccharides and their role in rhizosphere phosphorus mobilisation was further elucidated recently (Lidbury et al. 2021). In this study, we could further observe an increase of *Firmicutes* in the root samples (Figs S3 and S7). *Firmicutes* are frequently found in high numbers in paddy fields and they were shown to be one of the main players in the early anaerobic degradation of plant polymers (Wegner and Liesack 2016). Interestingly, investigating the distribution of *Firmicutes* in more detail revealed a niche separation between families of the *Bacilli* and *Negativicutes* on the plant roots, whereas *Clostridia* were abundantly present in root and soil samples (Fig. S7). Members of the *Peptostreptococcaceae* (*Clostridia*) were only abundant in soil samples. Overall, the increase of *Firmicutes* on plant roots was mainly based on an increase of *Bacilli* species. This large group of bacteria contains mainly aerobic species and also species with potential plant-growth-promoting activity have been described. Strains of the genera *Bacillus* and *Paenibacillus* for instance have been isolated from rice fields and their plant-growth-promoting activity was characterized (Beneduzi et al. 2008).

The archaeal communities in our study also differed in bulk soil and the rice roots. The roots were dominated by *Euryarchaeota* with hydrogenotrophic methanogens (such as *Methanobacteria* and *Methanocellales*) being highly abundant. The dominance of hydrogenotrophic versus acetoclastic methanogenesis on rice roots has been well documented and described before (Conrad 2007 and references therein). The soil samples were characterized by many sequences assigned to the *GOM Arch I* cluster which also comprises the anaerobic methanotroph *Methanoperedens*. In contrast to *ANME* clusters which couple methane oxidation to sulfate reduction and dominate in marine environments, *Methanoperedens* seem to be the most important anaerobic methane oxidizer in freshwater systems. Enrichment cultures have revealed a physiological flexibility of these archaea to use of nitrate, iron, manganese, and humic substances as electron acceptors (Ettwig et al. 2016, Cai et al. 2018, Bai et al. 2019, Leu et al. 2020). They seem to be the most abundant and active anaerobic methane oxidizers in paddy fields playing an important role in mitigating methane emission to the atmosphere (Vaksmaa et al. 2016, Vaksmaa et al. 2017, Fan et al. 2021). Furthermore, they seem to be remarkably resistant to oxygen stress (Guerrero Cruz et al. 2018). We also detected a lower, however, considerable number of *ANME-2a-2b* sequences in our study. Whereas *Methanoperedens* dominated the bulk soil samples, *ANME-2a-2b* was more abundant in root samples. To date, this group of methane oxidizing archaea has not been investigated in paddy fields, however, a recent study described sulfate-dependent anaerobic methane oxidation as minor pathway in Chinese paddy soil (Fan et al. 2021). In our study, potential syntrophic bacterial partners capable of sulfate reduction, such as *Syntrophobacteraceae*, were abundantly present in root samples and in earlier work, sulfate reduction rates were shown to be highest in rice root proximity (Wind and Conrad 1997).

Even though *Euryarchaeota* dominated in root samples, *Thaumarchaeota* were also found in high relative abundances in root as well as soil samples. They were identified as important nitrifiers in rice fields (Chen et al. 2008, Ke et al. 2013, 2014). A recent genome comparison revealed larger genomes in terrestrial as opposed to marine archaeal ammonia oxidizers (AOA) with potential adaptation strategies to soil including biofilm formation, detoxification, adhesion, and cell–cell recognition (Kerou et al. 2016). However, not much is known of the niche differentiation between the various terrestrial AOAs. In our study, we could observe a higher relative abundance of the *SCG* cluster (comprising *Nitrososphaera*) on the roots and the *SAGMCG-1* cluster (comprising the acidophilic *Nitrosotalea*) in soil samples.

*Bathyarchaeota* formed the third large group of Archaea in our data set. They are widespread and abundant in anoxic environments, both saline and freshwater. Although no cultures are available to date, genome sequencing and stable isotope labelling efforts suggest that they are heterotrophs involved in global carbon transformation (Zhou et al. 2018 and references therein). *Bathyarchaeota* are phylogenetically and physiologically diverse and among 25 subgroups, few might have a potential for methane metabolism (Evens et al. 2015). Our classification method does not allow a more detailed analysis of the proposed subgroups (Zhou et al. 2018), but further research on this thriving group of archaea in rice fields and their role therein is undoubtedly needed.

The rice cultivar did not have an effect on the microbial community composition in our study. Effects of the rice genotype have been shown in earlier studies, however, this effect was generally not very strong and could be observed in studies investigating the microbiota on the roots of genetically distant rice species, such as *O. sativa* (Asian rice) versus *O. glaberrima* (African rice), or *O. sativa japonica* versus *O. sativa indica* (Edwards et al. 2015, Santos-Medellin et al. 2017). The two cultivars in our study possess a different growth phenotype and belong to different mereological classes (Volante et al. 2017), however, both are *O. sativa japonica* cultivars and thus, might be too similar genetically to shape significant different root microbiomes.

### Effect of water management

Comparing the microbial alpha diversity under the different water managements, no strong or explicit pattern could be observed. Whereas the overall bacterial root diversity was not affected by the water treatment, the bulk soil bacteria and root archaea both showed a significant higher species richness under CF treatment. However, comparison of beta diversity clearly revealed different communities in the two water treatments. This effect was strongest for the microbial communities on roots and more explicit for bacteria than for archaea. Under regular water management, paddy soils are submerged during the rice growing season and drained again afterwards. This cycle of flooding and desiccation is repeated seasonally, in some areas even up to three times per year (Aulakh et al. 2001). Our study site in Vercelli (Italy) has a history of rice cultivation for over a century (Ferrero and Vidotto 2010) and thus, these soils have been exposed to many cycles of flooding and drainage. Over time, a soil community has been selected that is adapted to these conditions (Conrad 2020). It has been shown that microbial communities in rice fields stayed rather stable after drainage or re-flooding and even methanogenic archaea did not decrease considerably in numbers after desiccation and oxygen exposure (Breidenbach et al. 2016, Liu et al. 2018, Conrad 2020). This agrees with our study and the observation that the overall microbial composition in the bulk soil was only weakly affected by the different water management. However, the root communities were strongly affected and thus, may be more vulnerable to changes. The nutrient-rich and highly fluctuating environment surrounding the plant roots might select for fast growing microbial communities that also rapidly react to changing conditions. A similar observation has been made in an earlier study in which the microbial root communities were also stronger influenced by a different water management than the bulk soil communities (Chialva et al. 2020). Furthermore, in an experiment carried out at the same field site and using a highly similar plot arrangement as in our study, the root growth was shown to be affected by the two water treatments (Oliver et al. 2019). This further indicates drastically changing conditions for microorganisms on the plant roots in both treatments. However, the organic matter decomposition potential in the soil was similar under both water treatments. This is again in good agreement with our results showing that the bulk soil communities also did not change much under the different management.

The root microbiota reacting strong to the water management included the alphaproteobacterial families *Sphingomonadaceae*, *Rhizobiaceae*, and *Bradyrhizobiaceae* and the gammaproteobacterial *Enterobacteriaceae* and *Xanthomonadaceae*. Among others, these families also comprise many strains with plant-growth-promoting activities (Gopalakrishnan et al. 2015, Luo et al. 2019). In addition, they are often characterized by an aerobic lifestyle such as members of the *Sphingomonadaceae* which are aerobic hydrocarbon degraders (Kertesz et al. 2019). In contrast to the alpha- and gammaproteobacteria which were overall enriched under AWD treatment, families of the *Delta-* and *Epsilonproteobacteria* were depleted. This included anaerobic bacteria such as syntrophic fermenting, or iron and sulfate reducing bacteria of the *Geobacteraceae*, *Syntrophobacteraceae*, *Desulfovibrionaceae*, *Desulfobulbaceae* and *Cystobacteraceae*. Members of the *Syntrophobacteraceae*, *Desulfovibrionaceae*, and *Desulfobulbaceae* were identified as major propionate-dependent sulfate reducers in paddy soils (Liu and Conrad 2017). Knowledge on the role of *Epsilonproteobacteria* in the environment is still limited to date as research has mainly been focused on human and animal pathogens. However, hydrogen production of *Sulfurospirillum* species (*Campylobacteraceae*) upon fermentation and syntrophic interaction with a methanogen has been demonstrated recently (Kruse et al. 2018). Furthermore, *Sulfurimonas* species (*Helicobacteriaceae*) are important redox cycling organisms shown to couple the oxidation of sulfur compounds to the reduction of nitrate (Han and Perner 2015).

Besides *Proteobacteria*, families of the *Bacteroidetes* and *Firmicutes* were strongly affected by the different water management. The overall relative abundance of *Bacteroides* increased under AWD treatment, due to the increase of *Flavobacteriaceae*, *Sphingobacteriaceae*, *Cytophagaceae*, and *Chitinophagaceae*. These families comprise many characterized plant-growth-promoting bacteria and are usually aerobic hydrocarbon degrading bacteria (e.g. Madhaiyan et al. 2015, Chhetri et al. 2019). They can harbour a large potential to produce secondary metabolites (Figueiredo et al. 2021) which might be beneficial for the plant. In contrast, nearly all OTUs affiliated with the *Firmicutes* decreased in the AWD treatment, in particular families of the anaerobic *Clostridia*.

Of the archaea, *Euryarchaeota* and *Bathyarchaeota* decreased overall under AWD management, whereas the aerobic *Thaumarchaeota* increased in relative abundance. The strongest decrease could be observed for the *Methanobacteria* and *Methanocellales*, hydrogenotrophic methanogens which also belong to the dominant *Euryarchaeota* on plant roots. Interestingly, also the aerobic bacterial methane oxidizers (*Methylocycstaceae* and *Methylococcaceae*) decreased under AWD treatment, indicating less substrate availability and less total methane turnover.

## Conclusion

The use of more efficient water managements in rice agriculture is urgently needed to adapt to water scarcity and mitigate further climate change. Microorganisms play a key role in paddy fields as they are involved in nutrient cycling and greenhouse gas emission and can be important for plant health. In this study, we could observe that alternate wetting and drying compared to continuous flooding affected the microbial communities in paddy fields, in particular the root microbiota. Whereas the microbial communities in the bulk soil seemed well adapted and more resistant to changes in the water level, the root communities were vulnerable to the treatment. Here, many of the enriched microorganisms might be plant-growth-promoting bacteria, however, oxygen availability in the more aerated soils under AWD treatment seem to have the largest impact. Microorganisms with an overall anaerobic lifestyle were depleted in the AWD treatment whereas aerobic species increased in abundance. This was also in accordance with a decrease of microorganisms involved in methane cycling and might indicate a lower methane emission into the environment. Nevertheless, more aerated soils and aerobic degradation of organic matter might result in elevated CO_2_ emission. However, an earlier study has already shown that paddy fields under CF and AWD treatment both remained net CO_2_ sinks (Oliver et al. 2019). The Vercelli paddy fields investigated in this study are low in labile organic carbon (Oliver et al. 2019, Conrad 2020). Future research is needed to show if similar observations are also applicable for paddy fields with high organic carbon content.

## Supplementary Material

fiac018_Supplemental_Files
