# Supplementary material for: Effect of water management on microbial diversity and composition in an Italian rice field system
Source: FEMS Microbiol Ecol. 2022 Feb 16;98(3):fiac018. doi: 10.1093/femsec/fiac018 (PMC8924702; doi:10.1093/femsec/fiac018)
Supplement: fiac018_Supplemental_Files [file fiac018_Supplemental_Files.zip › Figure_S1.pdf]

AWD1

Baldo	Gageron	Gines
Vialone	Arelate	Llavar
Selenio	Puntal	Gleva
Centauro	Loto	Prometeo

CF1

Arelate	Llavar	Gleva
Vialone	Puntal	Gageron
Baldo	Centauro	Selenio
Loto	Gines	Prometeo

CF2

Puntal	Gageron	Llavar
Centauro	Gines	Vialone
Selenio	Prometeo	Baldo
Gleva	Arelate	Loto

AWD2

Gleva	Arelate	Centauro
Selenio	Vialone	Gageron
Llavar	Puntal	Loto
Prometeo	Gines	Baldo

AWD3

Baldo	Llavar	Puntal
Gageron	Gleva	Gines
Vialone	Selenio	Loto
Prometeo	Arelate	Centauro

CF3

Gageron	Loto	Vialone
Puntal	Gines	Selenio
Prometeo	Gleva	Llavar
Arelate	Centauro	Baldo

CF4

Centauro	Baldo	Llavar
Loto	Vialone	Selenio
Gageron	Arelate	Gines
Gleva	Puntal	Prometeo

AWD4

Llavar	Selenio	Centauro
Gines	Puntal	Prometeo
Gleva	Gageron	Baldo
Vialone	Arelate	Loto
