# Supplementary material for: Effect of water management on microbial diversity and composition in an Italian rice field system
Source: FEMS Microbiol Ecol. 2022 Feb 16;98(3):fiac018. doi: 10.1093/femsec/fiac018 (PMC8924702; doi:10.1093/femsec/fiac018)
Supplement: fiac018_Supplemental_Files [file fiac018_Supplemental_Files.zip › FigureLegendsSupplement_revision.rtf]

Figure legends supplement

Figure S1. Experimental design of the study. Field plots were cultivated under either continuous flooding (CF) or Alternate Wetting and Drying (AWD) water management. Plots were further divided into sub-plots planted with 12 European different rice cultivars in randomised order. In this study, samples were taken from plots planted with the rice cultivars 'Centauro' and 'Vialone Nano' (marked in different colour). From each plot, samples were taken in triplicates from bulk soil and plant roots, respectively (96 samples in total).

Figure S2. Alpha diversity of Bacteria (A) and Archaea (B) grouped according to plots planted with the two different rice cultivars. Indices: observed species richness (Observed), estimated species richness (Chao1), Shannon index (Shannon), and Simpson index (Simpson). The asterisk indicates significant differences between groups according to Wilcoxon rank-sum test (p<0.05, n.s.=not significant). Top: test between root and soil, Bottom: test between rice cultivars.

Figure S3. Relative abundance of the main bacterial phyla detected in soil and root samples under different water management (CF=Continuously flooded, AWD=Alternate Wetting and Drying). 

Figure S4. Relative abundance of the main archaeal phyla detected in soil and root samples under different water management (CF=Continuously flooded, AWD=Alternate Wetting and Drying). For the Eury- and Thaumarchaeota, further classification levels were shown in the order Phylum-Class-Order-Family-Genus. For Methanomassiliicoccus, the complete classification order was shortened to Phylum-Class-Genus.

Figure S5. Effect of water management on rare bacterial phyla in roots samples. A. Log2-fold changes of differentially abundant OTUs in AWD (Alternate Wetting and Drying) versus CF (Continuously Flooded) treatment (adjusted p-value cutoff=0.01). CF treatment was defined as control. OTUs were grouped on the family level. Circle sizes are correlated to the baseMean (mean of normalized counts for all samples) of the respective OTU. B. Mean relative abundances of total OTUs in root samples (n=17722) under AWD and CF treatment. 

Figure S6. Effect of water management on bacterial phyla in soil samples. A. Log2-fold changes of differentially abundant OTUs in AWD (Alternate Wetting and Drying) versus CF (Continuously Flooded) treatment (adjusted p-value cutoff=0.01). CF treatment was defined as control. OTUs were grouped on the family level. Circle sizes are correlated to the baseMean (mean of normalized counts for all samples) of the respective OTU. B. Mean relative abundances of total OTUs in soil samples (n=15781) under AWD and CF treatment. Phyla that include families with differential abundance as shown in A, are marked in the corresponding colour. Phyla that do not include differentially abundant families are marked in different shades of grey.

Figure S7. Differential abundance of Firmicutes in different field compartments (plant roots versus bulk soil). A. Log2-fold changes of differentially abundant OTUs in root samples versus soil samples (adjusted p-value cutoff=0.01). The soil compartment was defined as control. OTUs were grouped on the family level. Circle sizes are correlated to the baseMean (mean of normalized counts for all samples) of the respective OTU. B. Mean relative abundances of total OTUs assigned to Firmicutes (n=1649) in the different field compartments as well as under AWD and CF treatment. C. MA-plot showing total bacterial enriched and depleted OTUs in the different field compartments (total enriched: 1270, total depleted: 2660). 
