# Supplementary figures and images for: Effect of water management on microbial diversity and composition in an Italian rice field system
Source: FEMS Microbiol Ecol. 2022 Feb 16;98(3):fiac018. doi: 10.1093/femsec/fiac018 (PMC8924702; doi:10.1093/femsec/fiac018)

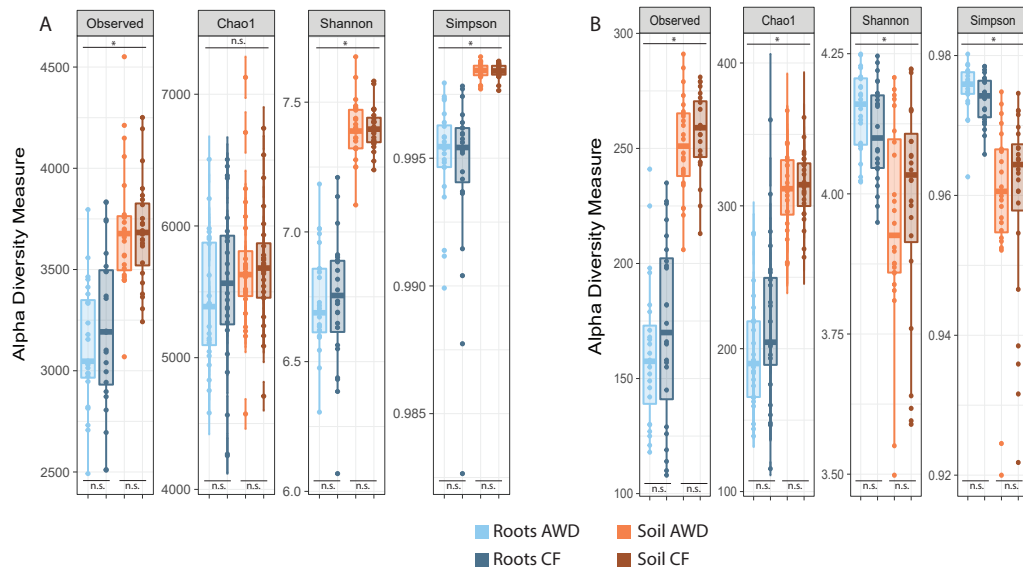

Supplement: fiac018_Supplemental_Files [file fiac018_Supplemental_Files.zip › Figure_S2.pdf]

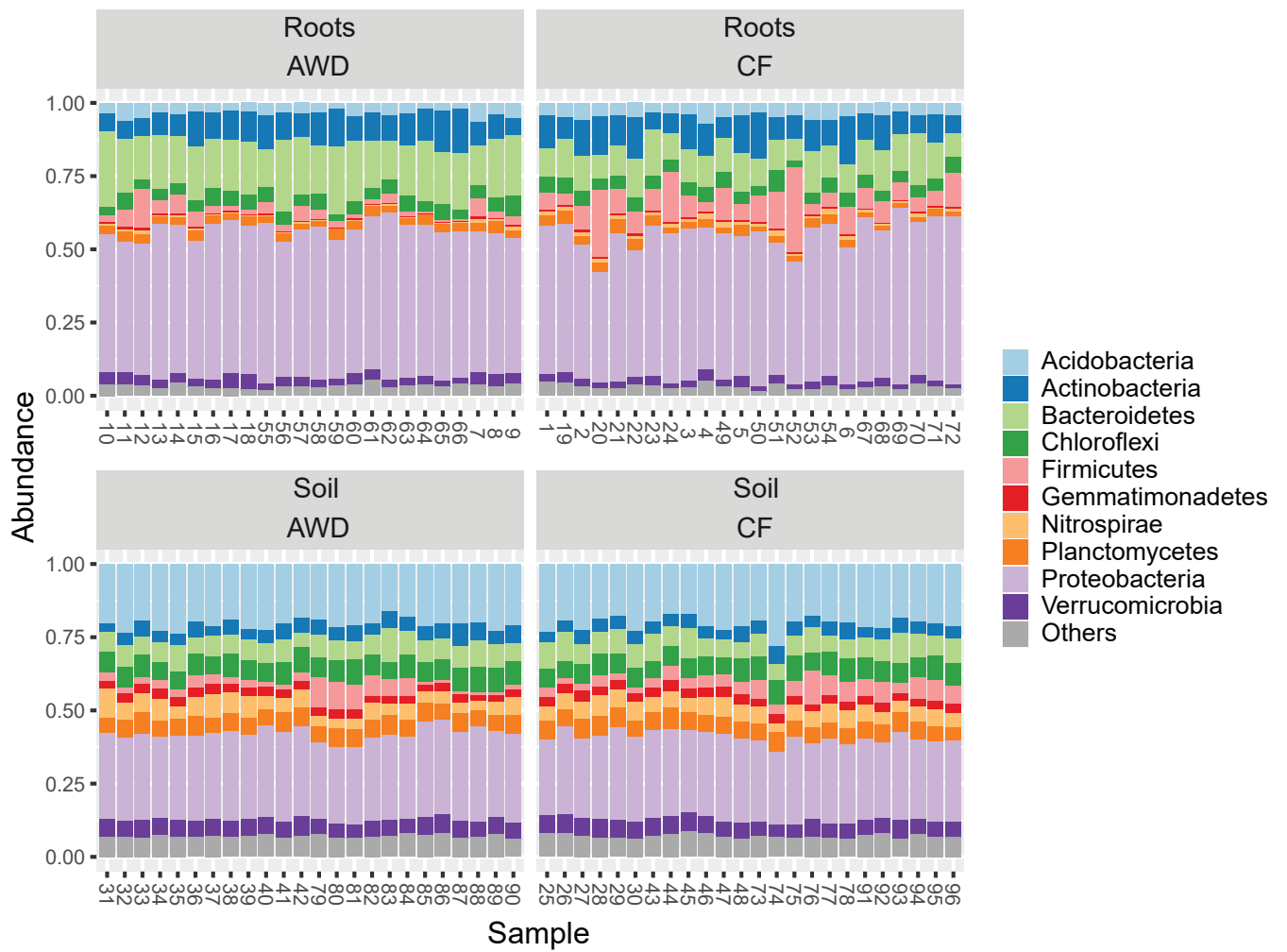

Supplement: fiac018_Supplemental_Files [file fiac018_Supplemental_Files.zip › Figure_S3.pdf]

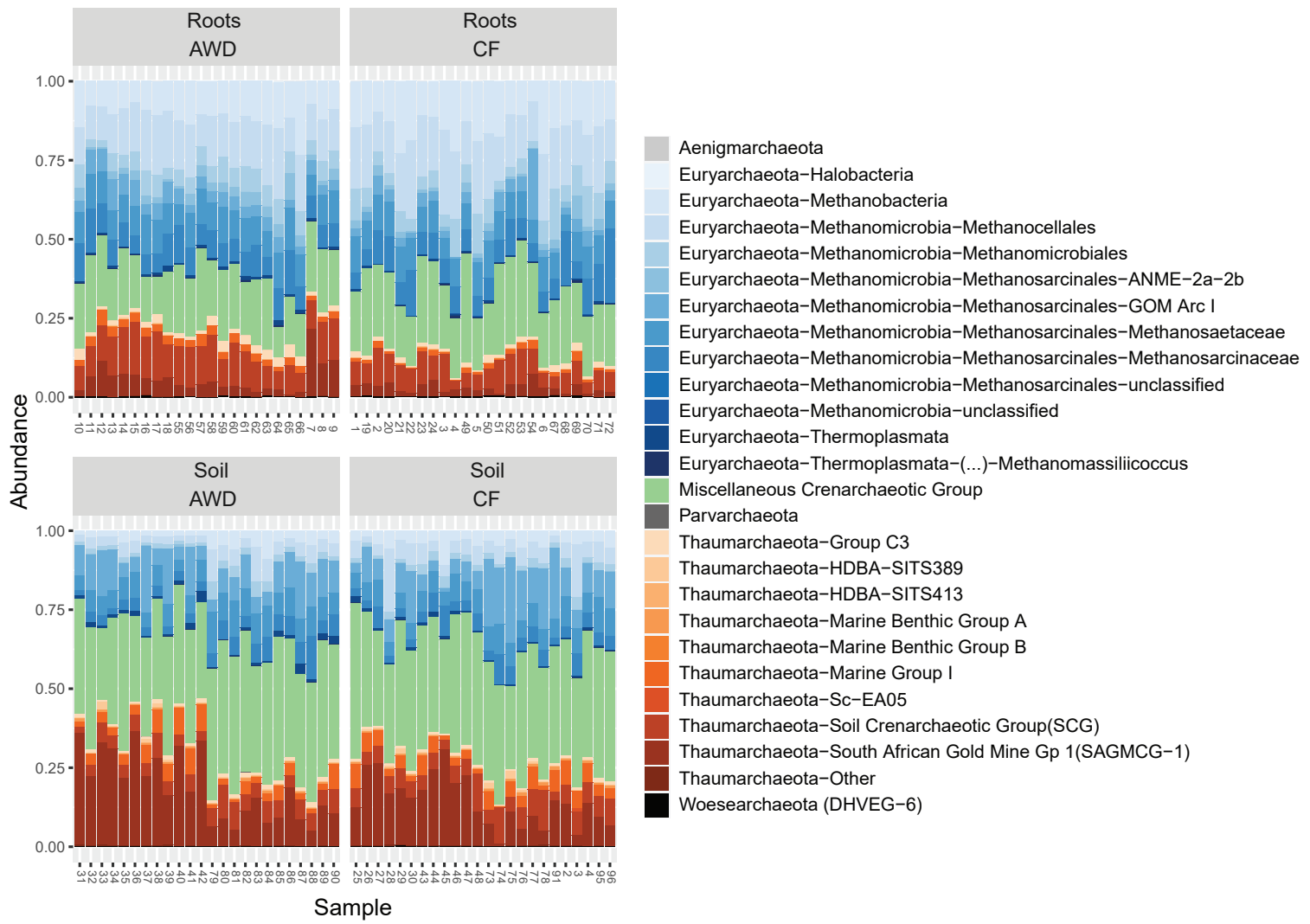

Supplement: fiac018_Supplemental_Files [file fiac018_Supplemental_Files.zip › Figure_S4.pdf]

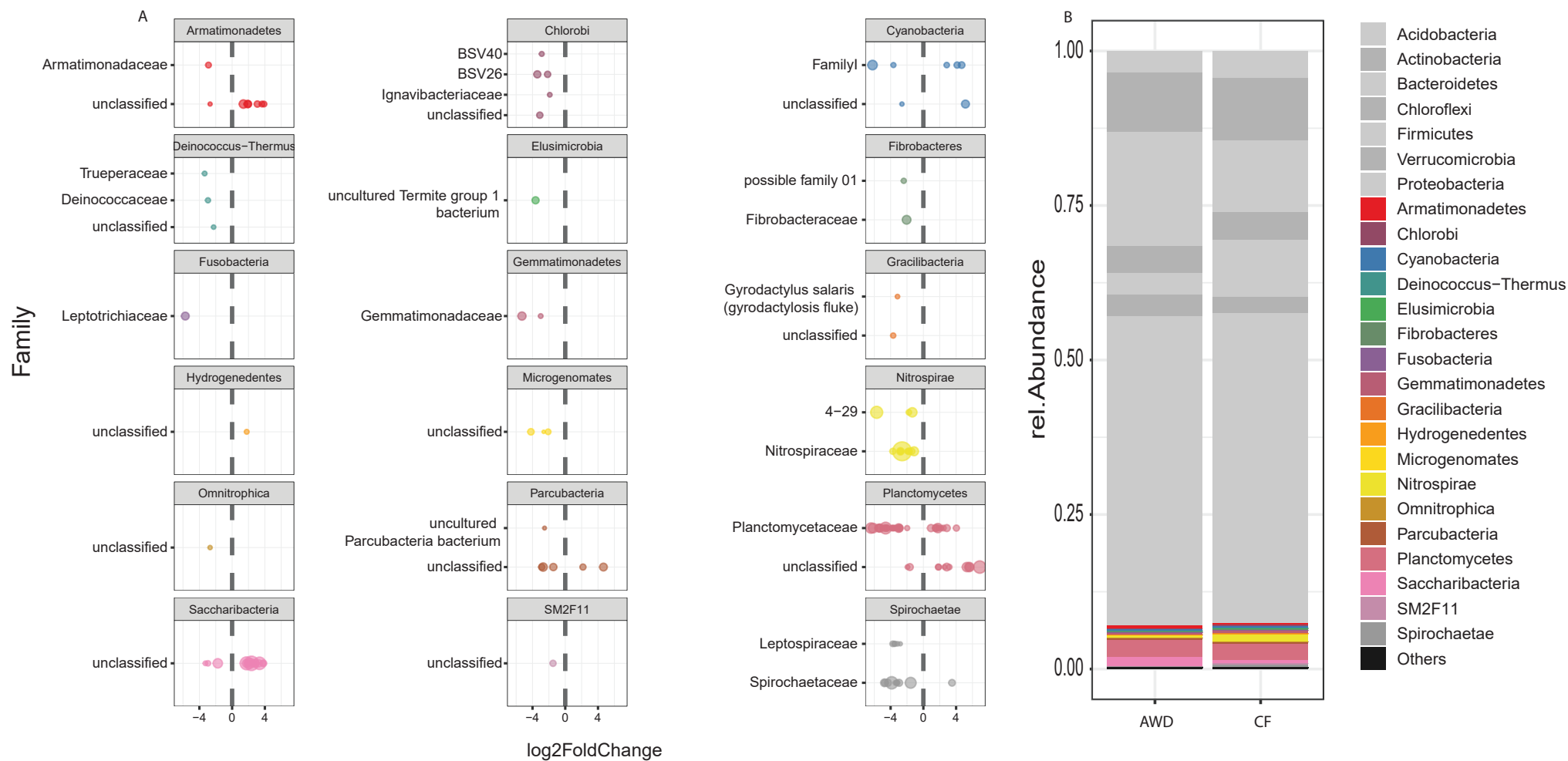

Supplement: fiac018_Supplemental_Files [file fiac018_Supplemental_Files.zip › Figure_S5.pdf]

A

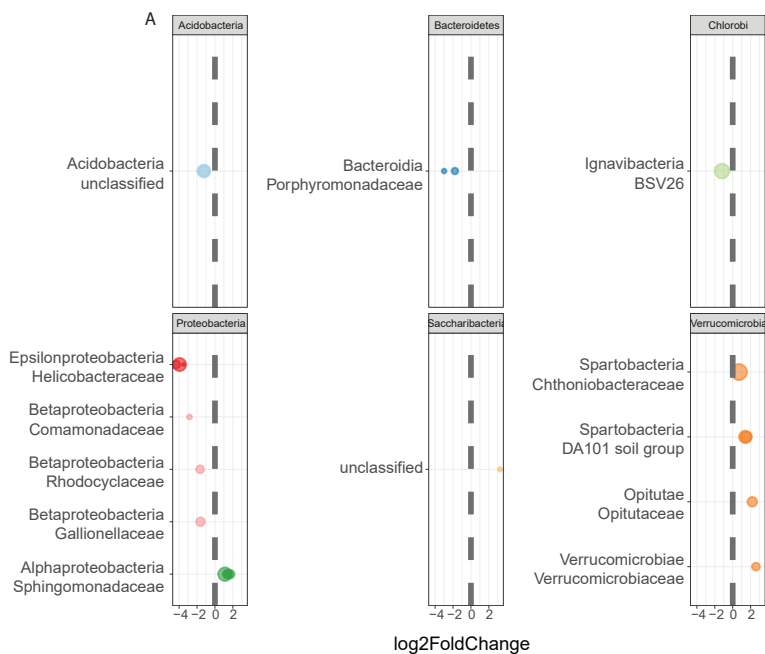

B

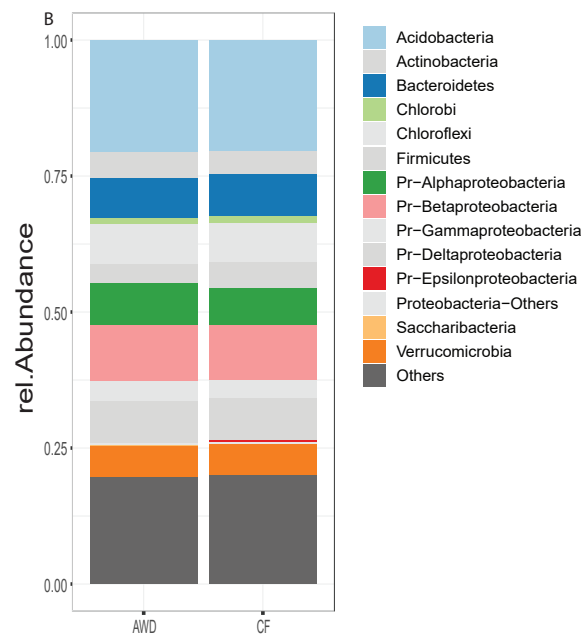

Supplement: fiac018_Supplemental_Files [file fiac018_Supplemental_Files.zip › Figure_S6.pdf]

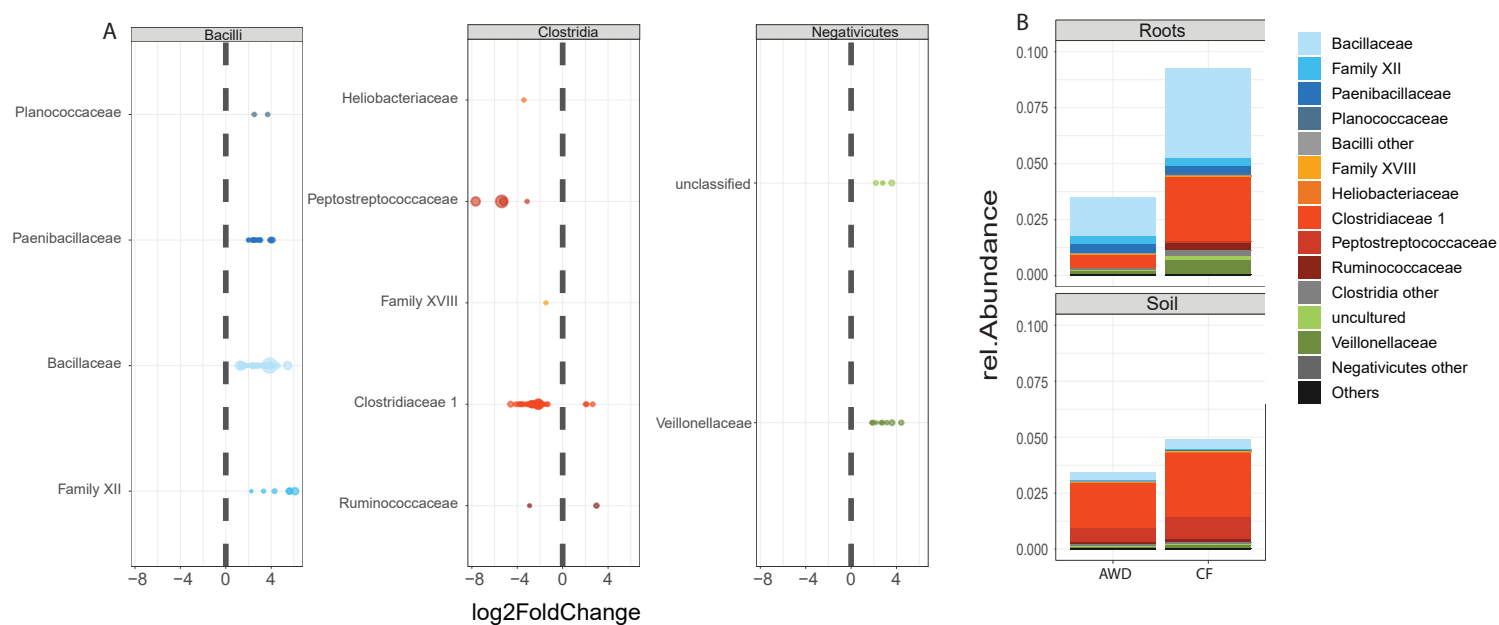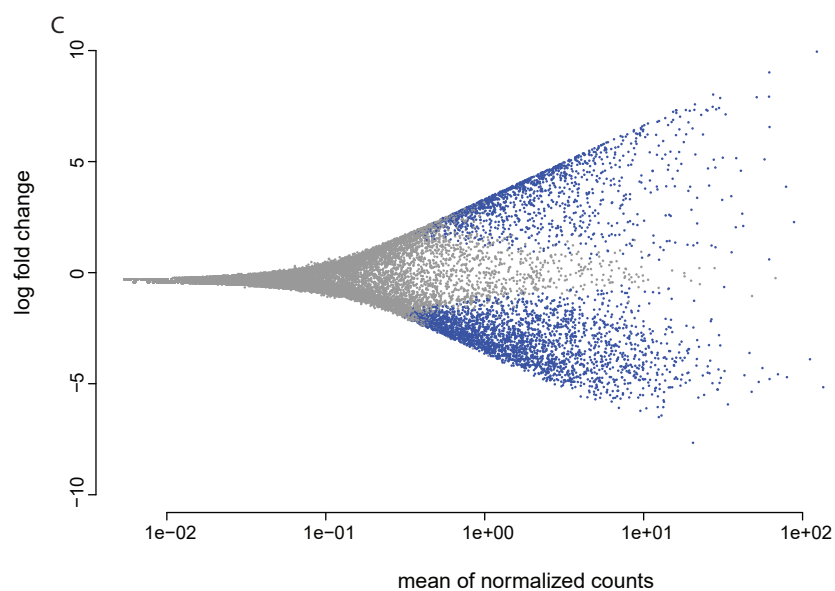

Supplement: fiac018_Supplemental_Files [file fiac018_Supplemental_Files.zip › Figure_S7.pdf]
